# Literature review of teaching skills programs for junior medical officers

**DOI:** 10.5116/ijme.5685.14da

**Published:** 2016-01-31

**Authors:** Jasan Dannaway, Heryanto Ng, Adrian Schoo

**Affiliations:** 1 Flinders University, School of Medicine, Sturt Road, Bedford Park, Adelaide, South Australia, Australia

**Keywords:** Junior medical officer, resident, teaching skills, residents as teachers, medical education

## Abstract

**Objectives:**

The aim of this review was to assess the current evidence regarding the efficacy of teaching skills programs for junior medical officers.  We aimed to compare and contrast these results with findings from previous literature reviews, the last of which were published in 2009.

**Methods:**

In order to capture studies since the last published literature reviews, five databases and grey literature were searched for publications from January 2008 to January 2015. A search for literature reviews without using the timeframe limitation was also performed.

**Results:**

The search from January 2008 to January 2015 resulted in the inclusion of 12 studies. Five systematic reviews of the topic were found which included 39 individual studies that were also analysed. Nearly all studies reported positive effects. Twenty nine studies reported change in attitudes, 28 reported modification in knowledge, 28 reported change in behaviour, 6 reported change in the organisation and two reported change in program participant’s students. There were substantial threats of bias present.

**Conclusions:**

The literature reviewed demonstrated many positive effects of teaching skills programs, which supports their utilization. However, high level outcomes need to be evaluated over longer periods of time to establish their true impact. An organisation specific approach to these programs needs to occur using sound course design principles, and they need to be reported in evaluation trials that are designed with robust methodology.

## Introduction

There have been a variety of organisational and educational challenges over the last few decades that have impacted on medical education. For example, student numbers are increasing, there is a drive toward evidence based education, and public expectations of a cost effective, responsible health service.^1^ This has created a complex struggle for clinical supervisors to balance teaching, research and clinical commitments.

Nearly 80% of final-year medical students in Australia who responded to a survey in 2011 demonstrated an interest in teaching.^2^ Junior medical officers (JMOs) (i.e. not specialists/consultants), spend a large proportion of their time teaching both students and their colleagues.^3^^-^^6^ Much of the “informal curriculum”, including professional values are taught by them when consultants are not around.^7^ Furthermore, residents who teach acquire taught content more effectively than by self-study or lecture attendance,^8^ and their teaching duties have been linked to greater job satisfaction.^9^ In the USA evidence of resident as teachers programs date back to the 1960’s^10^  and gained popularity in the 1990’s. In 1999 in the USA a survey of residency programs found that although residents provided 62% of hospital teaching, only 20% of the programs featured teaching skills programs.^11^ Literature from the USA has suggested that residents teach ineffectively. Studies have found that residents rarely cited literature, asked questions or gave feedback,^12^ were often insufficiently thorough,^13^ and generally did not cover problem solving skills, or psychosocial topics as often as consultants.^14^ Unfortunately, in many cases JMOs are expected to develop teaching skills as they proceed through training but often with little formal education on the topic.^4^^,^^15^ Various innovative programs have been developed in order to positively impact on residents as teachers. 

There have been some reviews of the topic.^16^^-^^22^ Of these reviews five^16^^,^^18^^,^^19^^,^^21^^,^^23^ have utilised systematic methods the last of which were published in 2009. Interestingly, although one was published in 2004 the others were published in 2008-2009. This may mean that there is overlap in their findings or that reviews have not analysed all available studies, particularly studies from 2008 onwards. Although these studies have generally supported the use of JMO teaching skills programs, the true effectiveness of these programs was unable to be established due to the lack of objective outcome measures and inadequate study designs.^19^ A number of recommendations and conclusions were reached by these reviews. Higher levels of evaluations, for example Kirkpatrick level 4 (Table 1) that measures the effect of the intervention in the real world,^24^ were called for. Longer follow up times were recommended in order to establish the longevity of courses effects. Unfortunately a literature review of this topic has not been performed in some time and there are inconsistencies in the conclusions of prior reviews which make interpretation of this topic difficult.

### Objectives

The aim of this review was to assess the current evidence regarding the efficacy of teaching skills programs for junior medical officers.  We aimed to compare and contrast these results with findings from previous literature reviews, the last of which were published in 2009, in order to provide up to date conclusions and recommendations regarding this topic

## Methods

### Search strategy and information sources

Medline, Embase, ERIC, CINAHL, and PsychINFO were searched from January 2008 to January 2015 by JD and HN. Grey literature, including that published by relevant medical bodies, was also utilised. Key words used were resident, registrar, teach, teacher, and education. Relevant papers were all those that had a focus of improving teaching skills of JMOs. Search results were screened by two authors JD and HN. The title and abstract of all results were screened. Full papers were then retrieved for further review if relevant. The references in these articles were also reviewed for further appropriate papers.

A similar search for systematic reviews on the topic without the timeframe limitation was performed to ensure that no studies were missed. Individual studies examined by the systematic reviews on the topic were also included in the analysis.

### Eligibility criteria

The following inclusion and exclusion criteria were developed for systematic reviews and individual studies.

Studies were included if participants were JMOs (i.e. not specialists/consultants). If they facilitated a structured teaching programme, detailing the teaching method and content. Included evaluation up to at least Level 2 of Kirkpatrick’s model. Non full text studies, not in English or those that included duplicated results were excluded.

### Data collection and analysis

A data extraction table was created in order to gather the required data for the review. Included studies were reviewed by JD and HN independently, and resulting data was synthesised. Study objective, context, method (study design, teaching intervention details, evaluation method), results (determined by Kirkpatrick’s levels of learning),^24^study quality and study outcome were retrieved.

Following independent data collection tables were merged into one and any discrepancies were resolved by consensus. In cases where consensus was not reached, a third reviewer (AS) determined the outcome.

 Kirkpatrick’s model of evaluation^24^ (Table 1) was used to categorise the outcomes reported by each of the included studies. The model outlines four progressively sophisticated levels of outcomes. Kirkpatrick’s model has existed for many years, is well known, logical, and also provides a convenient way to present outcome information.  It has been modified a number of times over the years and for the purpose of our review we have chosen to use the Best Evidence Medical Education (BEME) Collaboration^25^ version subsequently adapted by Hill et al. 2009^19^ to facilitate comparison of the literature.

Overall risk of bias was assessed for each included study. Two authors JD and HN independently evaluated bias using the critical appraisal worksheets for systematic reviews and therapy questions provided by the Centre for Evidence Based Medicine.^26^

**Table 1:** Kirkpatrick’s model for evaluating educational outcomes^19^

Kirkpatrick level	Evaluation outcome	Explanation
Level 1	Reaction	Participants’ views of the learning experience, its organisation, presentation, content, teaching methods, and quality of instruction
Level 2A	Learning – change in attitudes	Changes in attitudes or perceptions among participant groups towards teaching and learning
Level 2B	Learning – modification of knowledge or skills	For knowledge, this relates to the acquisition of concepts, procedures and principles For skills, this relates to the acquisition of thinking and problem-solving, psychomotor and social skills
Level 3	Behaviour – change in behaviours	Documents the transfer of learning to the workplace or willingness of learners to apply new knowledge and skills
Level 4A	Results – change in the system or organisational practice	Refers to wider changes in the organisation attributable to the educational programme
Level 4B	Results – change among the participants’ students and peers	Refers to improvement in medical student or peer learning or performance as a direct result of the educational intervention

## Results

The database search resulted in 6,373 papers. Following title and abstract review 59 papers were then retrieved for full text review. A hand search of the 59 papers reference list resulted in the retrieval of five more papers that were scrutinised. Those that did not meet inclusion criteria were excluded. The main reason for exclusion was the study did not describe a teaching skills intervention. All full texts could be retrieved for the review. Following this process a total of 12 of the 64 papers were included in the review.

In addition, seven literature reviews were found of which five were systematic that were included in the current study. The five systematic reviews together analysed 39 different papers. Hill et al.^19^ analysed 29 of these studies. The 10 remaining studies were included in one or more of the other reviews. Of these 10 papers four did not include any report on outcomes. There were no disagreements between the two authors regarding inclusion or exclusion.

### Characteristics of the studies

Of the 12 individual studies included, all but one was based in North America. One was based in New Zealand,^27^two in Canada^28^^,^^29^ and the rest in the USA (Table 2). A wide variety of specialties were included. Nine papers focused on one specialty.^28^^-^^36^ Five of which were Psychiatry.^28^^,^^30^^-^^32^^,^^36^ Studies included a range of clinical experience levels from postgraduate years 1 to 5. All studies stated the number of participants except one. The total number of included participants over all studies was 1,659, the minimum was 11, maximum 479 and the median was 78.

**Table 2:** Outline of studies after 2008

Author	Year	Overall risk of bias	Outcome Level*						Findings	Follow up duration
			1	2A	2B	3	4A	4B		
Hill	2012	Low			✓	✓		✓	Varied	1 year
Grady-Weliky	2010	Mod		✓	✓				Positive	1 day
Lehman	2010	Mod	✓	✓	✓				Positive	3 months
Ostapchuk	2010	Mod	✓	✓	✓	✓	✓		Positive	1 year
Donovan	2011	Mod	✓	✓					Positive	1 day
Wachtel	2013	Mod	✓	✓	✓	✓			Positive	1 day
Dang	2010	High	✓	✓					Positive	1 day
Daniels-Brady	2010	High			✓	✓	✓		Positive	2 months
Polan	2010	High	✓	✓					Positive	1 day
Pien	2011	High	✓	✓	✓	✓	✓		Positive	1 year
Ricciotti	2012	High	✓	✓	✓				Positive	4 years
Pernar	2013	High	✓	✓	✓	✓			Positive	1 year
			9	10	9	6	3	1		
			12	12	12	12	12	12		
Percentage			75	83	75	50	25	8		

*Kirkpatrick levels as described by Hill et al. 2009^19^

All studies outlined an aim. Most JMO teaching skills programs were established due to the large amount of resident teaching documented in literature or via internal audits and a lack of available teaching skills opportunities for JMOs. The specificity of study aims varied. Many included secondary objectives such as assessing resident self-reported efficacy.

There were no randomised controlled trials (RCTs). Two were non randomised controlled trials^27^^,^^34^ and the rest were uncontrolled trials. Only six studies included a pre intervention outcome measure.^27^^, ^^29^^, ^^31^^, ^^32^^, ^^35^^, ^^37^

There was a variety of follow up times. Six studies stopped follow up immediately after collecting data post workshop. Five studies included follow up to or greater than one year.^27^^,^^34^^,^^36^^-^^38^ The longest follow up was four years, the entire duration of the residency program.^33^

### Educational interventions and associated outcomes

Most program types detailed by the included studies were workshops. The shortest teaching program was 1.5 hours^29^ and the longest was 1.5 days.^27^ Seven included a single workshop as their intervention, whereas four delivered a series of workshops over time.

A variety of instructional methods were utilised. Most studies delivered content by mixed methods including role-play, brainstorming, reflection, and small-group discussion. Daniels-Brady et al.^30^ used direct supervision whereby the program director would meet the student weekly in order to advise them. Pernar et al.^34^ delivered content via emails on a weekly basis over the course of a year.

A variety of teaching content was delivered. Common topics included adult learning principles, feedback, reflection, curriculum orientation, and evaluation. Some authors utilised panels (including various stakeholders such as doctors and program directors) to design course content, while others utilised tools described in the literature. For example, Ostapchuck et al.^37^ utilised the previously validated^39^^,^^40^ Bringing Education and Service Together (BEST) curriculum (with some modifications). Daniels-Brady et al.^30^ utilised one on one, face to face individualised approach. The program director gave advice on teaching, curriculum of educational resources such as journal articles and observed residents giving feedback. Pernar et al.^34^ delivered brief statements (29 in total) via email of what constituted good teaching.

All studies included a survey as part of their evaluation.  Only two studies included alternative evaluation methods. For example, Ostapchuck et al.^37^ included focus groups of medical students, and Ricciotti et al.^33^ included a faculty assessed Observed Structured Teaching Evaluation (OSTE) (both videotaped and in person). All programs except one^41^ (who, instead, surveyed the students taught by them) sought the opinion of the JMOs. Faculty were also utilised to provide assessment of JMOs.

Individual studies published after 2008 showed that a range of Kirkpatrick levels were evaluated (Table 2). Ten studies reported level 1 outcomes, 11 reported level 2a outcomes and 10 level 2b, seven studies reported level 3 outcomes and four studies reported level 4a outcomes. One study reported level 4b outcomes. All studies reported positive findings. Five studies demonstrated positive outcomes in follow up more than or equal to 1 year.^27^^,^^34^^, ^^36^^-^^38^

Studies published prior to 2008 demonstrated that Kirkpatrick outcome levels were distributed similarly. Sixteen studies reported level 1 outcomes, 18 reported level 2a outcomes and 18 level 2b, 21 studies reported level 3 outcomes and two studies reported level 4a, outcomes. One study reported level 4b outcomes and five studies reported findings that were not positive (Table 3).          

**Table 3:** Outline of studies prior to 2008

Author	Year		Overall risk of bias		Outcome Level*												Findings		Follow up duration
					1		2A		2B		3		4A		4B				
Naji	1986		Low						✓		✓						Positive		Unclear
Edwards, Kissling and Brennan	1988		Low								✓						Positive		2.5 years
Dunnington	1998		Low		✓				✓		✓						Varied		6-7 months
Morrison	2003		Low				✓				✓						Positive		6 months
Morrison	2004		Low				✓				✓						Positive		6 months
D’eon	2004		Low								✓						Positive		6 months
Brown	1971		Mod		✓		✓										Positive		<1 month
Lawson	1980		Mod				✓				✓						Positive		3 months
Jewett	1982		Mod				✓		✓								Positive		6-12 months
Greenberg	1984		Mod				✓										Positive		9 months
Edwards	1986		Mod						✓								Positive		18-24 months
Edwards, Kissling and Plauche	1988		Mod		✓		✓		✓		✓						Positive		Unclear
Snell	1989		Mod				✓		✓		✓						Positive		8 months
Bing-You	1990		Mod				✓		✓								Varied		2-11 months
Katzelnick	1991		Mod		✓		✓		✓								Positive		8 months
Litzelman	1994		Mod								✓		✓				Positive		6 months
Roberts	1994		Mod				✓		✓				✓				Positive		6 months
Susman and Gilbert	1995		Mod		✓				✓								Positive		<1 month
Spickard	1996		Mod				✓		✓		✓						Varied		8 months
Bing-You	1997		Mod		✓		✓		✓								Positive		12 months
Barth	1997		Mod						✓								Positive		Unclear
Wipf	1999		Mod						✓								Positive		3 years
Furney	2001		Mod		✓				✓		✓						Positive		1 month
Mass	2001		Mod						✓								Positive		Unclear
Thomas	2002		Mod		✓		✓								✓		Positive		6 weeks
Frattarelli and Kasuya	2003		Mod		✓		✓										Negative		6 months
Pandachuck	2004		Mod		✓						✓						Positive		Unclear
Hammoud	2004		Mod		✓						✓						Positive		9 months
Busari	2006		Mod		✓						✓						Positive		3-4 months
Gaba	2007		Mod		✓		✓				✓						Positive		6 months
Rubak	2008		Mod						✓		✓						Positive		6 months
Aiyer	2008		Mod		✓		✓		✓		✓						Positive		Unclear
White	1997		High		✓						✓						Positive		10 weeks
Jafri	2007		High		✓		✓				✓						Positive		1 month
Moser	2008		High								✓						Positive		At 6 and 9 months
					16		18		18		21		2		1				
					39		39		39		39		39		39				
Percentage					41		46		46		54		5		3				
																			

*Kirkpatrick levels as described by Hill et al. 2009^19^

### Appraising and weighting the evidence

Individual studies published after 2008 showed substantial threats to their internal validity. Of the uncontrolled trials only five included pre and post intervention outcome measures and none of the non-randomised controlled trials did so. Some studies had small samples sizes. Six studies had more than 10% loss to follow up which was not consistently explained. Only two papers used validated (or variations of) surveys and objective outcome measures.^27^^,^^33^ There was response bias present in some studies as participation was voluntary and thus likely to attract those who were keen to teach.

Five literature reviews of varying quality were included in the analysis. The study by Wamsley et al. 2004^16^ was the earliest detected review in this area. The review only searched Medline for studies between 1975 and 2003. Fourteen papers were included in the analysis. There was no bias risk assessment performed, and there was some heterogeneity in the results found.

Dewey and colleagues^18^ conducted a systematic review of trials with an interest in gathering information on how to develop teaching skills in psychiatry residents. They searched a number of databases and 13 studies were identified and analysed. Methodological quality and study characteristics were quantified, but they did not specify the period by which they searched.

Hill and colleagues^19^ performed a review to determine the characteristics of effective teaching skills programs. They searched a number of databases from 1971 and 2008, and performed a bias risk assessment of the 29 included studies.

Lacasse et al.^21^ conducted a systematic review of resident teaching with a focus on family medicine. They identified eight studies from 1950 to 2008 (they only searched two databases and have missed studies) that all included family medicine residents. They did not perform a bias risk assessment of included studies.

Post et al.^23^ reported on 24 studies from 1975 to 2008 that looked at programs that were designed to improve resident teaching skills. Although they provided a descriptive review of these studies, they did not include an individualised risk to bias of the studies included in their review. They did quantify aspects of study design.

Studies published prior to 2008 of which were included in the five literature reviews demonstrated many threats to validity. That said the quality of these was generally more robust than that of the studies published after 2008. There were 12 RCTs. There were nine non-randomised controlled trials and 14 uncontrolled trials. The methodological quality of the RCTs varied. Randomisation methods, concealment of randomisation, group differences at baseline and blinding of raters were poorly reported and adjusted for, although this is difficult to do with RCTs of this topic. Only three studies had over 10% loss to follow up.^42^^-^^44^ Only four studies did not utilise objective outcome measures.^44^^-^^47^ Of the non-randomised controlled trials all included pre and post intervention outcome measures. Three used validated, reliable and objective outcome measures.^48^^-^^50^ Seven of the uncontrolled trials included complete pre and post intervention outcome measures.^51^^-^^56^ Six used validated and reliable outcome measures.^10^^,^^41^^,^^54^^-^^57^ Seven studies utilised objective outcome measures.^52^^, ^^54^^, ^^55^^, ^^57^^-^^59^

## Discussion

This review has made an assessment of the current evidence regarding the efficacy of teaching skills programs for junior medical officers. These results have built upon findings from previous literature reviews, the last of which were published in 2009.

Consistent with previous literature reviews, studies regularly demonstrated a positive impact on perceptions and attitudes towards teaching (Kirkpatrick level 2a).  Positive impact was also demonstrated supporting modification of knowledge or skills (Kirkpatrick level 2b) Although substantial bias is present in most studies, which prevents definite conclusions regarding the real impact of teaching programs on developing JMOs teaching skills (Kirkpatrick level 3), the results are more likely to be positive than negative. Improving student learning (Kirkpatrick level 4b) through teaching programs was only investigated by one study after 2008, who did not prove benefit.^19^ Studies did demonstrate that positive organisational change could occur due to the intervention (Kirkpatrick level 4a). One study created a teaching resident position that enabled the dual benefit to the resident and the students that the resident was teaching.^30^ Another created a “train the trainer” program which enabled the dissemination of teaching skills courses across the organisation.^38^ Ostapchuck et al. found that their teaching skills program led to invitations for the delivery of the program to other areas of the organisation, became a point of difference to attract residents to their program and gained interest from the education board.^37^ Level 4b changes, are difficult to evaluate and are seldom reported. One study Hill et al 2012^27^ implemented a 1.5 day workshop to 34 interns which demonstrated improvement using the intern clinical teaching effectiveness instrument when compared to control hospitals.  However, objective structured clinical examination results of medical students taught by these interns, did not demonstrate any significant intersite differences. Although in this setting there was no difference found this study demonstrated an innovative method to assess this outcome.

### Evidence for teaching skills programs

There were five systematic reviews identified through the search with a combined total of 39 studies that were analysed. Hill et al.^19^ analysed 29 of these studies. The six studies^39^^,^^45^^,^^47^^,^^51^^,^^52^^,^^60^ that they did not include have added further information although do not change the overall findings of their review. We therefore feel that the review by Hill and colleagues represent well the literature prior to 2008.

The individual analysis of all 39 studies demonstrates clearly that there is convincing evidence that supports the efficacy of JMO teaching skills courses in improving Kirkpatrick outcomes up to level 3. Six studies had a low risk of bias. The most well-constructed studies were those by Morrison et al.^39^^,^^40^ who also generally reported positive outcomes up to and including level 3. One of the six studies^53^ found results that may not be sustainable, which indicates the ongoing need for longer detailed follow up in this area. There were 12 randomised controlled trials of varying quality. When these studies where analysed as a group they reveal similar findings as discussed above.

Twelve studies published after 2008 were identified that reported outcomes regarding teaching skills programs for JMOs.  This group consists of studies with heterogeneous designs, programs, and outcome measures. All studies reveal useful insights, although it is difficult to quantify the effects of programs on teaching skills of JMOs. There is significant evidence that teaching skills programs have subjective positive effects on all Kirkpatrick outcome levels. Unfortunately, what is not present is strong evidence for objective positive effects for higher levels of outcomes (Kirkpatrick levels 2b, 3 or 4) and their effects over time. In this sense these studies have not been able to add more evidence to this important topic.

### Implications for designing and delivering teaching skills programs for JMOs

Literature reviews to date have aimed to find the aspects of a JMO teaching skills program that make it effective. To date this has proven difficult due to the heterogeneity of programs reported. The evidence so far suggests that looking for a common “formula” may be a flawed approach, but that the incorporation of individualised teaching skills initiatives into the JMO curriculum is likely a more effective approach. Detailing this process is beyond the scope of this article but the following are some suggestions.

Important components of curriculum design include a situational analysis, statements of intent (aims, objectives, outcomes), content, implementation and organisational strategies, assessment, and monitoring and evaluation all need to be considered (not necessarily in any particular order). One helpful guide is Susan Toohey’s (1999) “course design process”^61^ that suggests a sequence of events for the design process. Adult learning principles should be considered at all points during the design process.^62^ Finally, the Kirkpatrick’s model of evaluation is a good way to conceptualise, design and report evaluation. Evaluation methods, ideally, need to make use of valid and reliable outcome measures such as the Personal Teacher Identity Scale, OSTE, Clinical Teaching Assessment Form, Stanford Faculty Development Program Form-26, Clinical Teaching Effectiveness Instrument or the Resident Leadership Scale to establish the effect of programs on teaching skills of JMOs and their impact on those who they teach.

### Limitations

There were some limitations to our review. Included studies were largely heterogeneous in their methods and chosen interventions. All studies but one were conducted in North America.  A large number were conducted at single institutions and only involved one discipline, which makes generalizability of results difficult.

### Implications and recommendations for further research

Unfortunately, we have demonstrated that studies on this subject have substantial methodological flaws. We do however appreciate the difficulties involved with designing the ideal trial. From a methodological perspective future trials should ideally be prospective randomised controlled trials. These trials should be designed to avoid issues with internal validity. This includes pre and post intervention assessment, valid randomisation methods with concealment, blinding of assessors if applicable and the use of validated, reliable and objective outcome measures. Also, these trials should use a standardised process of reporting outcomes (we suggest the Kirkpatrick model), so that their results can be easily interpreted and compared with other studies. They should focus on determining the effects of programs on high Kirkpatrick’s levels including 2b, 3, 4a, 4b and whether the effects are long lasting.

## Conclusions

 In reviewing the literature on teaching skills programs for JMOs the findings of published literature reviews and individual studies have been considered and appraised. Teaching skills are important for JMOs to possess.  Longer follow up times are needed to establish the impact of these programs in the real world. Since medical programs vary, no set “formula” for these educational programs can be applied. An individualized approach should occur with sound course and research design principles followed.

### Conflict of Interest

The authors declare that they have no conflict of interest.
